# Impact of early palliative care intervention in autologous bone marrow transplantation: feasibility of a multicentric study

**DOI:** 10.1186/s12904-024-01499-z

**Published:** 2024-07-25

**Authors:** María Argüello-Marina, Marta Callejas-Charavía, Beatriz Merchán-Muñoz, Daniel Gainza-Miranda, Agustina Rico-Zampetti, Raquel Pérez-Maganto, Gustavo Ruiz-Ares, Patricia García-Ramírez, Dunia de Miguel-Llorente, Julio García-Suárez

**Affiliations:** 1 Hematology Department, Hospital Universitario de Guadalajara, Guadalajara, Dunia de Miguel, Spain; 2 Hematology Department, Hospital Universitario Príncipe de Asturias, Alcalá de Henares, Madrid, Spain; 3 Palliative Care Department, Hospital Universitario Príncipe de Asturias, Alcalá de Henares, Madrid, Spain

**Keywords:** Autologous haematopoietic stem cell transplantation, Palliate care intervention, Quality of life

## Abstract

**Introduction:**

This prospective multicentre study evaluates the impact of Palliative Care Unit (PCU) intervention (Experimental Group, EG), during autologous hematopoietic stem cell transplantation (AHSCT) on quality of life (QoL), symptom control and healthcare resource use compared to standard practice (Control Group, CG). We used validated scales on Days 0 (stem cell infusion), + 7 (bone marrow aplasia, acute symptoms) and + 21 (aplasia recovery).

**Results:**

In 40 patients (20 EG/ 20 CG: 45%/25% female, median age 57.5/59), QoL differed significantly at Day + 7 (EG: median 0.50; CG: -63.00; *p* < 0.001) and Day + 21 (EG: -2.00; CG: -129.00; *p* < 0.001). On Day 0, mean FACT-BMT scores were CG/EG: 131/ 89.35, reflecting the pre-transplant intervention of the PCU in EG patients. For pain (EG median 0.00, CG median 2.50; *p* = 0.01), 45% EG patients used opioids on day 0 (mean 38.5 mg morphine/day/patient). Reduced pain control impacted nutritional support (parenteral nutrition 45% CG, 5% EG; *p* = 0.08). Hospitalisation duration was longer in CG (median 18.5; EG median 13.00; *p* < 0.001). Despite the short follow-up and small sample size, PCU and HD collaboration improves QoL and symptom management during acute AHSCT, evident through pain control, analgesia management, reduced parenteral nutrition need and shorter hospital stays.

## Introduction

The haematological patient undergoing Autologous Haematopoietic Stem Cell Transplantation (AHSCT) is a complex case. Indeed, the conditioning chemotherapy regimen, integral to the transplantation process, is linked to varied and occasionally severe symptomatology [1]. Additionally, severe neutropenia resulting from the cytostatic treatment requires reverse isolation, which has a notable psychological impact on the patient, often underestimated by clinicians [2].

Palliative care (PC) teams adopt a multidisciplinary approach focusing on comfort and quality of life (QoL) of patients and their relatives (https://www.who.int/health-topics/palliative-care). In recent years, several manuscripts have been published showing that PC intervention in patients with haematological malignancies appears to improve physical and emotional symptoms control, foster hope, QoL, and may reduce caregiver burden [3–7]. However, further studies are needed to analyse the outcomes of PC intervention.

A recent study led by El-Jawahri A. et al. [8–10] explored the potential of early palliative care unit (PCU) intervention in the management of haematological patients undergoing haematopoietic transplantation. The results demonstrated that a multidisciplinary approach may enhance the QoL of patients by improving symptom control, reducing emotional impact, minimising post-traumatic stress, and lessening the burden on caregivers. The authors also emphasised the necessity for additional studies to compare these results with a control group and to assess the impact of PC team interventions.

Research in PC is challenging due to ethical issues, barriers between medical specialties, and poorly defined standards of care for palliative patients. Moreover, the substantial symptom burden complicates the completion of questionnaires, hindering patients’ participation in clinical studies [11–13].

The main objective of this study is to assess the feasibility of conducting a prospective, multicentre, non-randomised, non-blinded study comparing symptomatic control in patients undergoing AHSCT in a hospital with a protocolised early intervention of PCU versus standard intervention. The Al-Jawari study was conducted at a single centre, and we aim to demonstrate that the study can be carried out between two centres with similar characteristics, one of which does not have a PCU. Furthermore, in Spain, there is no established collaboration policy between Hematology and Palliative Care services. Access to these teams by oncohematological patients is infrequent and generally delayed, so patients are not familiar with the scales used or the support services provided by this service.

The secondary objectives include analysing the impact of early PCU intervention in terms of symptomatic control, functionality, QoL, and length of hospitalisation.

## Methods

### Population

The experimental group (EG) hospital (UHPA) provides healthcare services to around 243 000 inhabitants, mainly in urban areas, but also in rural areas in the northeast of Madrid. The control group (CG) hospital (UHG) covers 250 000 inhabitants, both in rural areas and in the city of Guadalajara itself; it is also the reference hospital for AHSCT in two provinces of Castilla la Mancha (Ciudad Real and Cuenca). The two hospitals are university-affiliated and share similar characteristics in terms of size, staff, and range of services; and they are only 20 km apart.

The study included all consecutive patients undergoing AHSCT who met the inclusion criteria: age ≥ 18 years, signed informed consent and no significant cognitive impairment or language barrier, from March 2021 to June 2022. Patients hospitalised in the UHPA were included in the EG, while the CG consisted of patients of the UHG, given that this hospital did not have a hospital PCU.

All patients signed an informed consent form before their inclusion in this study.

### Study design

This is a pilot feasibility study with a limited number of participants to assess the viability of the protocol and obtain additional information on the required sample size.

**Fig. 1:**
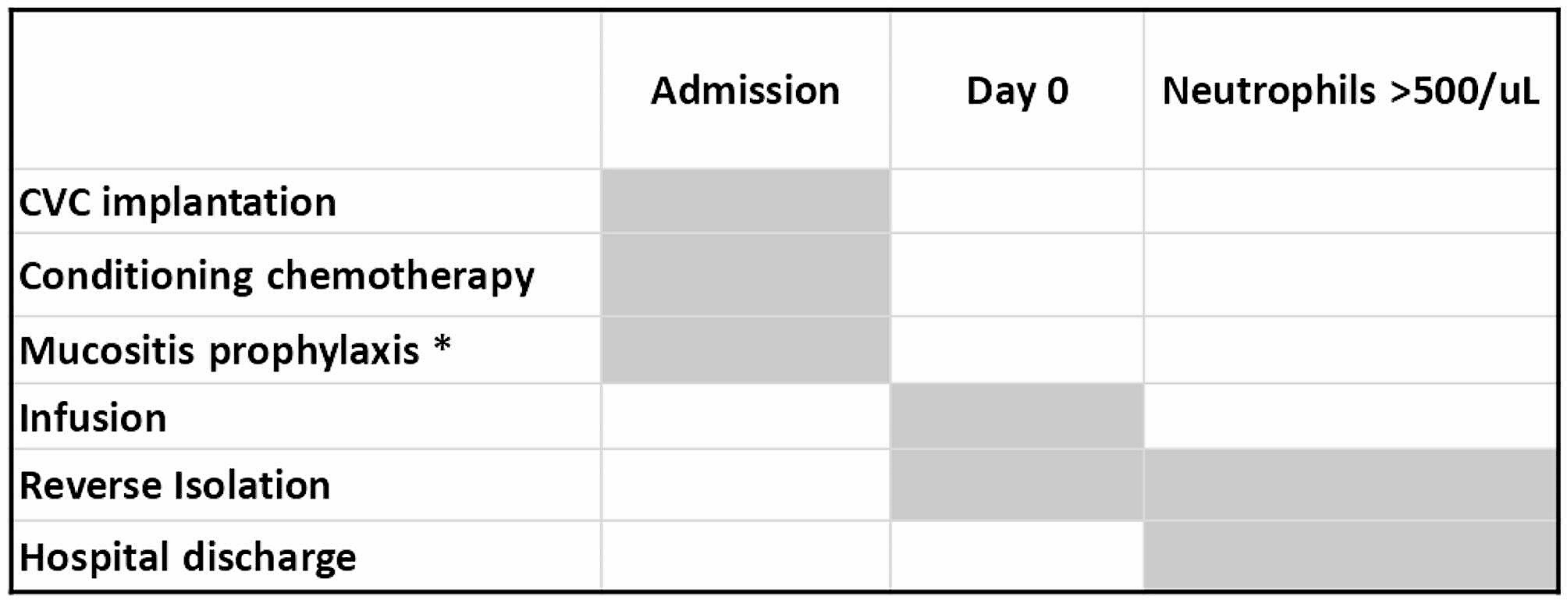
AHSCT protocol AHSCT: autologous haematopoietic stem cell transplantation. CVC: central venous catheter. * The mucositis prophylaxis protocol consisted of cryotherapy during the administration of melphalan, as well as administration of Ectoin mouthwash in the case of EG patients

In both groups, AHSCT was conducted with a similar protocol as described in Fig. 1.

In addition, in the EG, the PCU of the UHPA (formed by 3 doctors, 3 nurses, a psychologist and a social worker) conducted daily visits to the patient during admission, followed by a re-evaluation after 21 days in the outpatient clinic.

Throughout these visits, the PCU systematically assessed the symptom burden at each stage of the procedure: on Days 0 (stem cell infusion), + 7 (bone marrow aplasia, acute symptoms) and + 21 (aplasia recovery), adjusting the treatment based on the observed symptoms or signs. The clinical psychologist, following an initial assessment, scheduled regular visits tailored to the individual needs of each patient and their caregivers. Lastly, an evaluation of the main caregiver’s situation was performed. If the main caregiver experienced care overload, the hospital social worker assessed the family situation and proposed personalised support measures.

### Study variables

To facilitate a comprehensive comparison of intervention outcomes, the following variables were evaluated in both study arms:

#### Socio-demographic

Age, sex, history of anxious-depressive syndrome, baseline comorbidities and prior treatments leading up to AHSCT were collected from the electronic medical records.

#### Symptom burden

Assessment of symptom burden was conducted using the revised Edmonton Symptom Assessment System (ESAS), which measures 10 symptoms on a 0–10-point scale. Higher scores indicate a higher symptom burden [14]. Anxiety and depression levels in patients were gauged using the 14-item Hospital Anxiety and Depression Scale (HADS), which consists of two subscales assessing symptoms of depression (HADS-D) and anxiety (HADS-A), with scores ranging from 0 (no distress) to 21 (maximum distress). Cut-off scores above 7 indicate clinically significant symptoms [15].

#### Functionality

Patient functionality was assessed using the Palliative Performance Scale (PPS) [16] and the Barthel Scale [17, 18]. Both scales, scored from 0 to 100, ascertain the degree of patient dependency and autonomy. A Barthel score below 20 indicates total dependence, while a PPS of 0% signifies death and 100% represents full functionality.

#### Quality of life (QoL)

The Functional Assessment of Cancer Therapy-Bone Marrow Transplant (FACT-BMT) [19] was employed to evaluate patients’ QoL. Comprising 47 items across four domains—physical well-being, functional well-being, emotional well-being, and social well-being—higher scores indicate improved QoL. Additionally, a fifth domain, “additional concerns,” (23 items) addresses specific aspects related to transplantation.

#### Primary caregiver overload

The reduced Zarit Scale determined the existence of primary caregiver overload, with a score ≥ 17 indicating familial claudication [20].

#### Study protocol summary

**Fig. 2:**
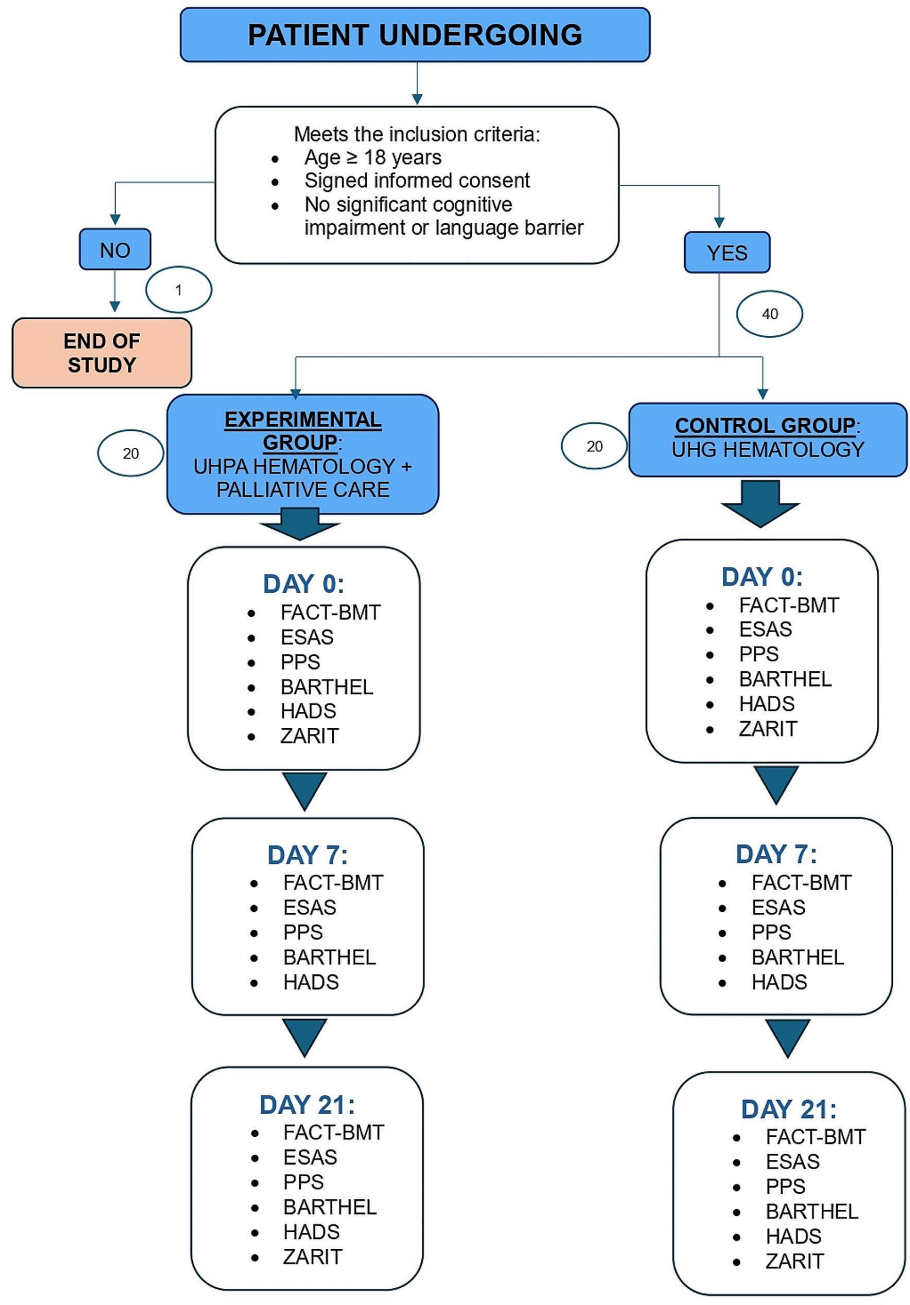
Visual representation of the study protocol

Refer to Fig. 2 for a visual representation of the study protocol.

#### Ethical approval

The study received approval from the ethics committee of the Hospital Principe de Asturias on 26/02/2021, with the code PAL-TASPE.

### Sample size calculation

To calculate the sample size, we estimated an effect size of 0.60 standard deviations (SD), with a statistical power of 80% and a significance level of 0.05. Considering a dropout rate of 20%, it was determined that at least 20 patients per group were needed to detect significant differences between the groups.

### Statistical analysis

In the analysis of demographic parameters, qualitative variables were analysed using Fisher’s exact test (2 × 2 matrices) or chi-square test (matrices larger than 2 × 2). Quantitative variables were analysed using the Wilcoxon test. For the analysis of variables collected at different time points, Kruskal-Wallis (KW) tests were used. In these analyses, the response variables used were those obtained by subtracting the value of each variable at 7 or 21 days from the value corresponding to the same patient at baseline. All statistical analyses were performed using the R statistical package (R Core ed, 2020).

### Results

Forty-one patients were included in the study: 20 from the CG cohort and 21 from the EG cohort. One patient in EG was excluded due to language barrier (in the EG). Patients´ characteristics of both cohorts were comparable in percentage of males and females, age of patients, background, frequency of patients with different baseline diagnoses, conditioning regimens, frequency of patients with different lines of treatment and risk associated with the transplant process, calculated by Hematopoietic cell transplantation - specific comorbidity index (HCT-CI). (Table 1).

**Table 1:** Sociodemographic and medical characteristics

	**CG†**	**EG‡**	p-valor*
**Sex**			0.20
Female	9 (45%)	5 (25%)	
Male	11 (55%)	15 (75%)	
**Background (rural/urban)**	14/6	2/18	**<0.01**
**Age (in years)**	57.50§ (16.00)	59.00† (12.25)	0.40
**Hematological Malignacy**			0.20
AL amyloidosis	0 (0%)	1 (5%)	
Monoclonal gammopathy of renal significance (MGRS)	0 (0%)	1 (5%)	
Diffuse large B cell lymphoma (DLBCL)	1 (5%)	1 (5%)	
Plasma cell leukemia (PCL)	1 (5%)	0 (0%)	
Hodgkin Lymphoma (HL)	4 (20%)	2 (10%)	
Mantle cell lymphoma (MCL)	3 (15%)	1 (5%)	
T-cell Non-Hodgkin Lymphoma (T-NHL)	2 (10%)	1 (5%)	
Anaplastic large cell lymphoma	0 (0%)	1 (5%)	
Multiple myeloma	9 (45%)	12 (60%)	
POEMS syndrome	0 (0%)	1 (5%)	
**Treatment line**			0.90
1ª	14 (70%)	13 (65%)	
2ª	5 (25%)	6 (30%)	
3ª	1 (5%)	1 (5%)	
**HTC-CI score**			0.60
Low risk	16 (80%)	13 (65%)	
Intermediate risk	2 (10%)	4 (20%)	
High risk	2 (10%)	3 (15%)	
**Conditioning Regimen**			0.45
Melphalan 200	10 (50%)	11 (55%)	
Melphalan 140	0 (0%)	4 (20%)	
BEAM	10 (50%)	5 (25%)	

†Control Group; ‡Experimental Group; §Median (IQR)

*Pearson’s Chi-squared test; Wilcoxon rank sum test; Fisher’s exact test

All patients who underwent AHSCT during the study period, agreed to participate and all of them completed the study. No patients died during the study period.

QoL according to the FACT-BMT scale showed a statistically significant difference between cohorts at day 7 (X2KW = 20.67; *p* < 0.001). The total FACT-BMT value was significantly higher in the CG (median: 0.50; interquartile range (IQR): 10.75) than in the EG (median: -63.00; IQR: 128.25), and the difference was maintained at 21 days (X2KW = 29.28; *P* < 0.001): EG (median: -2.00; IQR: 17.75); CG (median: -129.00; IQR: 63.50). On day 0, the mean FACT-BMT in the CG was 131 and in the EG 89.35 (*p* < 0.001). Thus, the differences obtained in the FACT-BMT results between the CG and the SG were 41.65 on day 0, 20.75 on day 7 and 43.4 on day 21. The results obtained on the FACT-BMT Scale on days 0, 7 and 21 are summarised in Table 2.

**Table 2:** Mean FACT-BMT† scale on days 0, 7 and 21 in control group (CG) and experimental group (EG)

	Day 0 (*p*<0,001)	Day +7 (*p*<0,001)	Day + 21 (*p*<0,001)
**UHG (CG)‡**	131	112.8	131.15
**UHPA (EG)§**	89.35	92.05	87.75

† FACT-BMT: Functional Assessment of Cancer Therapy-Bone Marrow Transplantation; ‡ UHG: (control group); § UHPA: (experimental group)

Regarding symptomatic control, we found a significant difference in pain scale values between CG and EG patients (X2KW = 5.95; *P* = 0.01), being significantly higher in the CG cohort (median: 2.50; IQR: 3.00) than in the EG cohort (median: 0.00; IQR: 1.75). This significance disappeared after 21 days (X2KW = 0.02; *P* = 0.89), after the acute phase of the process. 45% of patients in the EG were using opioids on day 0 (mean 38.5 mg of morphine/day/patient), while none of the patients of the CG were under opioids. This difference was maintained throughout the admission, with 100% of patients in the third step of Analgesic Ladder belonging to the EG, versus the CG, with 95% of patients (*p* = 0.001).

No statistically significant differences were obtained in the rest of symptoms assessed with the ESAS Scale (Table 3).

**Table 3:** ESAS† scale results (median) for control group and experimental group on days 0, +7 and +21

	UHG (control)			UHPA (Experimental)			*p*-value		
	Day 0	Day +7	Day + 21	Day 0	Day +7	Day + 21	Day 0	Day +7	Day + 21
**Pain**	1.55	4.5	2.15	1.3	2.35	1.75	0.33	**0.01**	0.89
**Tiredness**	3.45	5.8	4.85	3.3	5.2	4.65	0.96	0.66	0.73
**Drowsiness**	2.5	4.55	2.55	3.3	4.5	3.3	0.37	0.62	0.73
**Nausea**	2	3.15	1.25	2.45	2.5	1.45	0.35	0.27	0.99
**Lack of appetite**	3.25	5.6	3	4.3	5.3	3.4	0.17	0.25	0.42
**Shortness of Breath**	0.3	1.15	0.65	0.6	2.3	1.4	0.37	0.4	0.31
**Depression**	2.3	3.65	3.05	1.95	2.55	1.75	0.86	0.2	0.25
**Anxiety**	2.95	3.4	1.7	2	2.95	1.9	0.41	0.9	0.29
**Night Rest**	2.8	4	2.7	3.25	2.95	2.65	0.35	0.1	0.73
**Well-Being**	3.1	3.85	3.15	3.25	3.9	3.15	0.53	0.42	0.46
**Diarrhoea**	1	3.9	1.45	0.8	4.1	1.15	0.76	0.98	0.58

†ESAS: Edmonton Symptom Assessment System

Also, no statistically significant results were obtained in terms of functionality, measured by the Barthel and PPS scales, and the HADS anxiety and depression scales.

Primary caregivers identified as at risk of overload by the reduced Zarit Scale (3 in the EG and 2 in the CG) were assessed by the social worker in both groups. The differences were not statistically significant.

Finally, a significant difference was observed in the number of total days of admission between CG and EG patients (X2KW = 24.3; *p* < 0.001), being significantly higher in CG patients (median: 18.5; IQR: 2.25) than in EG (median: 13.00; IQR: 2.00).

## Discussion

Our results show that the study is certainly feasible given the high percentage of protocol completion and 0% dropout rate. Furthermore, the data suggest that the involvement of a PCU improves pain control, QoL and reduces the average length of stay of patients undergoing AHSCT.

In the PC field, there are many barriers and difficulties in conducting experimental studies. Typically, the high symptom burden, referring to both physical and emotional distress, makes it difficult to complete questionnaires [12]. On the other hand, the widespread concept of PC intervention in end-of-life care may be a barrier to implement early intervention in patients receiving intensive treatment with curative intent. Furthermore, in the specific case of haematology, several specific barriers to referral to PCU have been described. These included a lack of need for referral to PCU, difficulty in identifying referral time, negative perception by patients of PCU as end-of-life care, lack of clear prognostic indices to support the referral decision, and little communication between different Medical Departments [21].

As an example of this, a systematic literature review published in 2012 explained that the close relationship between hematologists and patients, developed during patient follow-up, makes referral on intervention by Palliative Care specialists challenging. Physicians often perceive their role solely as “treating the disease process,” leading to a fear of abandoning the patient [22]. Thus, we are facing a generalised situation of complicated work collaboration rooted in conflicts that may arise from early PC referral, the feeling of abandonment, the challenge of defining palliative care for hematological patients, and the lack of a collaborative culture between the two services.

However, in this study, the high completion rate among study participants shows the viability to respond to an unmet need for multidisciplinary management in a procedure with a high symptom burden such as AHSCT.

The results related to symptomatic control and QoL suggest that the joint intervention of the PCU and the Haematology Department improve the results compared to the CG, particularly in pain control. A clinically meaningful difference in pain control and quality of life is a noticeable and relevant change that improves the patient’s well-being and functionality, going beyond statistical significance.

This improvement in pain control and, consequently, QoL, is also observed in two studies published by El-Jawahri et al. on a transplant population, although the populations aren’t strictly comparable, as patients underwent both autologous and allogeneic transplantation [9, 23]. Within haematology, another study published by Porta-Sales and Guerrero-Torrelles supports this improvement in pain control, analysing early PCU intervention in patients with MM. They describe a reduction in pain prevalence from 24% in the first visit to 2% in the third visit for the population diagnosed with MM [5]. However, early PCU intervention is more developed in oncology, with several studies reporting improved pain and QoL after early PC intervention [24–27].

Focusing in the results of this study, we emphasise that, already on day 0, a lower symptomatic burden of pain than expected was observed in both groups. This could be explained by a higher percentage of lymphomas in the CG, while in the EG myelomas were the most common ones and 55% were already under opioids for pain control, as they are managed jointly with PC in outpatient clinics from the diagnosis. Multiple myeloma (MM) is a pathology in which pain is a central and very significant symptom, as observed in studies by Porta, where the use of opioids for pain control is similar to our study [22]. The increased use of opioids is also reflected in Porta’s study analysing early PCU intervention in patients with MM, where patients in outpatient follow-up shared with the PCU increased opioid use by 35.8% between the first and third visit [5]. This is an important point, as the patient admitted with a better general condition and less symptomatic burden, both physical and emotional, generally respond better throughout the process. This also facilitates the acceptance of early intervention by the PCU during hospitalisation. These results could be comparatively analysed in a larger-scale study with a sub-analysis of a patient cohort, stratifying according to underlying disease and symptom burden at the time of admission.

Particularly in terms of QoL outcomes, using the methodology of Osoba et al. [28], which correlates numerical results of statistical significance on QoL scales to their clinical relevance, we can categorise our results according to the following magnitudes: small changes (5 to 10 points), moderate changes (10 to 20 points), and large changes (> 20 points). Therefore, we can conclude that the difference in QoL between CG and EG in our study is large at all time points measured.

Also, we found no significant differences in anxiety and depression as measured by the HADS anxiety-depression scales, in contrast to El-Jawahri et al. study of 2016, where a significant impact is observed at 2 weeks, with a decrease in the anxious-depressive symptom burden [8]. The small sample size of our study could impact this result, and also the lack of statistical significance in functional assessment results (Barthel and PPS scales). A larger sample would probably achieve statistical significance.

Likewise, in terms of caregiver overload, as per El-Jawahri et al. [8], we also found no significant differences between the two groups, possibly due to the short follow-up time to complete a social intervention, as well as less participation of the main caregiver in the CG (probably related to stricter measures for visiting patient in isolation, complicating contact with patient by the responsible physician) and limited access to social resources in both centres. In another study by El-Jawahri et al., focused on patients with lung cancer, there is a decrease in anxious-depressive symptoms in the primary caregiver as measured by the HADS scale in the group of patients with early PCU intervention [29].

In conclusion, the study design and methodology are feasible. The intervention of a PC team seems to improve symptomatic control, especially pain and QoL, and reduces hospitalisation time.

### Limitations of the study

The study has some limitations. Firstly, the small sample size may condition some of our results. However, the main objective of the feasibility study does not seem to be limited by this situation.

Secondly, the study population has undergone autologous transplantation, so our results may not be generalisable to the population undergoing allogeneic transplantation.

We consider another limitation of the study to be the lack of an experimental and control group in both hospitals, but this was not possible due to the absence of a Palliative Care Unit (PCU) in control group.

Another potential limitation is that there is heterogeneity in baseline patient characteristics between the two study populations: socio-economic differences have not been analysed and higher percentage of patients with lymphoma in control group versus plasmatic cell dyscrasias in experimental group.

## Conclusion

This study is feasible since all patients agreed to be included and complete the study.

Further studies are needed to confirm that the PCU intervention appears to improve symptomatic control and quality of life (QoL) in patients undergoing AHSCT, and it even seems to decrease the length of hospitalisation. Conducted in a different healthcare system and cultural context from previously cited studies, our research highlights the consistency of our results with existing literature, underscoring the reproducibility and reliability of our study. This further validates the effectiveness of palliative care interventions while underlining the potential of early palliative care intervention during the HSCT process to foster relationships and build trust with patients. Our findings suggest that integrating palliative care early in the treatment process can effectively enhance patient outcomes, supporting the continued advancement of palliative care both in clinical practice and research.

## Data Availability

Data is provided within the manuscript,
